# Cichoric Acid May Play a Role in Protecting Hair Cells from Ototoxic Drugs

**DOI:** 10.3390/ijms23126701

**Published:** 2022-06-16

**Authors:** Ting-Wei Lai, Hsin-Lin Cheng, Tzu-Rong Su, Jiann-Jou Yang, Ching-Chyuan Su

**Affiliations:** 1 Department of BioMedical Sciences, Chung Shan Medical University, Taichung 402, Taiwan; w010941@gmail.com (T.-W.L.); iamsamlee@livemail.tw (H.-L.C.); 2 Antai Medical Care Corporation Antai Tian-Sheng Memorial Hospital, Pingtung 928, Taiwan; a085085@mail.tsmh.org.tw; 3 Department of Beauty Science, Meiho University, Pingtung 912, Taiwan; 4 Department of Medical Research, Chung Shan Medical University Hospital, Taichung 402, Taiwan

**Keywords:** cichoric acid (CA), zebrafish, hair cell, anti-oxidant

## Abstract

Ototoxic hearing loss due to antibiotic medication including aminoglycosides and excess free radical production causes irreversible hair cell injury. Cichoric acid, a naturally occurring phenolic acid, has recently been found to exert anti-oxidative and anti-inflammatory properties through its free radical scavenging capacity. The present study aimed to investigate the protective effects of cichoric acid against neomycin-induced ototoxicity using transgenic zebrafish (*pvalb3b*: *TagGFP*). Our results indicated that cichoric acid in concentrations up to 5 μM did not affect zebrafish viability during the 2 h treatment period. Therefore, the otoprotective concentration of cichoric acid was identified as 5 μM under 2 h treatment by counting viable hair cells within the neuromasts of the anterior- and posterior-lateral lines in the study. Pretreatment of transgenic zebrafish with 5 μM of cichoric acid for 2 h significantly protected against neomycin-induced hair cell death. Protection mediated by cichoric acid was, however, lost over time. A terminal deoxynucleotidyl transferase dUTP nick-end labeling (TUNEL) assay and FM4-64 staining, respectively, provided in situ evidence that cichoric acid ameliorated apoptotic signals and mechanotransduction machinery impairment caused by neomycin. A fish locomotor test (distance move, velocity, and rotation frequency) assessing behavioral alteration after ototoxic damage revealed rescue due to cichoric acid pretreatment before neomycin exposure. These findings suggest that cichoric acid in 5 μM under 2 h treatment has antioxidant effects and can attenuate neomycin-induced hair cell death in neuromasts. Although cichoric acid offered otoprotection, there is only a small difference between pharmacological and toxic concentrations, and hence cichoric acid can be considered a rather prototypical compound for the development of safer otoprotective compounds.

## 1. Introduction

Ototoxic injury refers to the functional dysregulation of the auditory and vestibular systems and is accompanied by damage to hair cells, which eventually leads to hearing loss [1,2,3]. The sensory hair cells located in the inner ear and lateral line system of zebrafish exhibit high genetic sequence conservation and structural morphology similar to those of humans, suggesting that zebrafish might be an appropriate model for studying sensorineural hearing loss [4,5]. The genetic tractability, high fecundity, and optical transparency of zebrafish also make them excellent tools for investigating human disorders. Thus, zebrafish larvae have recently become an emerging behavior model in determining inner ear relay sensory transduction of hearing and balance [6]. Accumulating studies have indicated that the ototoxic action of aminoglycosides is mainly due to reactive oxygen species (ROS) generation, which exacerbates oxidative stress and is strongly implicated in the apoptosis and necrosis of hair cells [7,8,9]. Notably, the reported prevalence of aminoglycoside-induced ototoxicity varies from 2% to 25% among patients, which is likely to be underestimated [10]. Nevertheless, aminoglycoside antibiotics are still widely used due to their low cost and antibacterial activity despite the availability needs prescription [11]. *In vitro* and *in vivo* evaluations of pharmaceutical prevention and the amelioration of ototoxicity from aminoglycoside antibiotics have been performed on a compound-screening platform to identify potential candidates that can improve drug-induced ototoxic hearing loss [12,13,14]. Herein, neomycin, the most ototoxic aminoglycoside, was employed as an inducer in the model of drug-induced hearing impairment in zebrafish.

Cichoric acid (CA), a plant phenolic compound derivative from caffeic acid, was first identified in *Cichorium intybus* and is also a major ingredient in *Echinacea purpurea*, which was recently found to exert a wide spectrum of pharmacotherapeutic effects, especially anti-viral, anti-oxidative stress, anti-hyperglycemic, and anti-obesity properties [15,16,17]. In a mice model of alcohol-induced acute hepatic steatosis, oral administration of CA (4 mg/kg body weight) for four consecutive days before alcohol administration remarkably protected against acute ethanol ingestion caused liver damage [18]. Diao et al. analyzed the CA metabolic profile from plasma, urine, and feces of rats by using ultra-performance liquid chromatography/quadrupole time-of-flight mass spectrometry methods. After oral administration of CA (100 mg/kg body weight), 19 metabolites of CA were determined [19]. Similar results were found in a pharmacokinetics study from a plasma sample after oral intake of *Echinacea purpurea* extract [20]. As previously described, CA significantly activates the antioxidant enzymes and, thus, the potential therapeutic values of CA for humans are worth further investigation [21,22]. Pellati et al. evaluated the free-radical scavenging capacity of phenolic extracts from *Echinacea* using spectrophotometric assay, showing that CA exhibited high anti-oxidation function through its DPPH radical scavenging ability [23]. CA also has anti-tumor actions owing to its anti-proliferative capacity, as it suppresses telomerase activity and β-catenin expression and activates caspase-9-dependent apoptotic signal transduction [24]. Furthermore, the in vitro protective effects of CA have been demonstrated, in which the substance serves as an effective ROS scavenger to reduce type III collagen degradation in xanthine or xanthine oxidase-induced free radical damage [25]. These previous studies have described the effectiveness of CA in attenuating or preventing disease-associated inflammatory and oxidative status, which underlines its potential in clinical therapeutic applications of hearing loss.

As hair cells in lateral line neuromasts in zebrafish are similar to those within the human inner ear, we previously developed transgenic zebrafish (*pvalb3b*: *TagGFP*) for investigating sensory hair cell loss as an in vivo testing platform for drug-induced ototoxicity [26]. In the present study, we aim to verify the otoprotect role of CA on neomycin-induced hair cell damage in transgenic zebrafish larvae and further explore the regulatory effects of CA on adaptive behavior through the sensory-motor coordination of larvae zebrafish swimming performance.

## 2. Results

### 2.1. The Dose-and Time-Response Testing of CA on Transgenic Zebrafish Larvae

**Figure 1:**
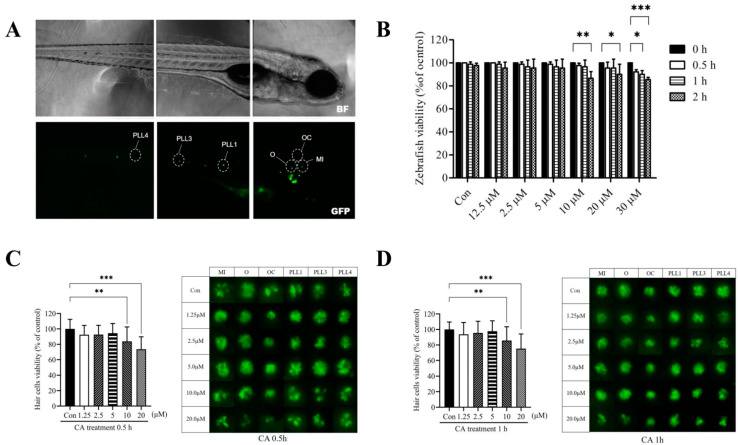
Effects of CA on transgenic zebrafish larvae and hair cell viability. (**A**) Fluorescence micrograph of a 7-dpf transgenic zebrafish larvae. The analyzed lateral line hair cells in zebrafish are marked by a white circle: otic (O), occipital (OC), middle (MI), and posterior lateral line (PLL1, PLL3, and PLL4). Scale bar: 100 μm. (**B**) 7-dpf transgenic zebrafish larvae were treated with CA (0, 1.25, 2.5, 5, 10, 20, and 30 μM) for different time (0.5, 1, and 2 h). After the exposure period, viable fish were counted and presented as a percentage of the untreated control. (**C**) Lateral line hair cells were treated with CA (0, 1.25, 2.5, 5, 10, and 20 μM) for 0.5 h and (**D**) 1 h. Fluorescence micrographs of lateral line hair cells from neuromasts (O, OC, MI, PLL1, PLL3, and PLL4) (right panel) were analyzed and the quantitative result of viable hair cells was present as a percentage of the untreated control (left panel). All values of the experimental groups were presented as mean ± SD. * *p* < 0.05, ** *p* < 0.01, and *** *p* < 0.001 as compared with untreated control. CA, CA; O, otic; OC, occipital; MI, middle; and PLL, posterior lateral line.

For the acute toxic experiment, a short-time exposure test was performed to determine the cytotoxicity of serial concentrations (0, 1.25, 2.5, 5, 10, 20, and 30 μM) of CA. The 7-dpf transgenic zebrafish larvae were treated with the indicated concentrations of CA for 0.5 h, 1 h, and 2 h. As shown in Figure 1, the viability of transgenic zebrafish larvae in the presence of 0–20 μM CA at 0.5 h and 1 h showed no significant difference compared to the 0 h time point (*p* > 0.05) (Figure 1B). A remarkable decrease in viability was observed in larvae treated with 10 μM (2 h), 20 μM (2 h), and 30 μM (1 and 2 h) of CA (*p* < 0.05).

### 2.2. Cytotoxicity of CA on Hair Cells in Neuromasts

To determine the cytotoxic effect of CA on hair cells, the effects of serial concentrations of CA on hair cell numbers in the neuromasts within the anterior otic (O), occipital (OC), middle (MI), and posterior-lateral regions (PLL1, PLL2, and PLL3) (Figure 1A) in transgenic zebrafish were assessed after 0.5 and 1 h treatment. As shown in Figure 1C,D, the number of hair cells in the presence of both 10 and 20 μM CA was significantly lower than in the untreated control after 0.5 and 1 h treatment, respectively (*p* < 0.05). However, CA concentration ranging from 0–5 μM did not affect hair cell viability after 0.5 and 1 h treatment (*p* > 0.05). The results suggested that CA concentrations up to 10 μM might potentially protect against drug-induced hair cell damage.

### 2.3. Protective Effect of CA Concentrations on Neomycin-Induced Hair Cells Loss in Transgenic Zebrafish Larvae

**Figure 2:**
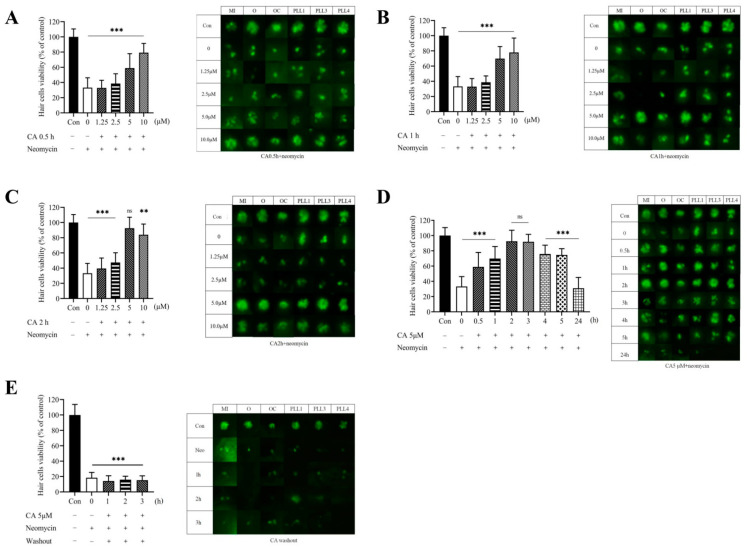
CA protected against neomycin-induced lateral line hair cell loss. (**A**) Transgenic zebrafish larvae were fixed and photographed using a fluorescence microscope. 7-dpf transgenic zebrafish larvae were treated with 12.5 μM of neomycin only for 0.5 h or pre-treated with CA (0, 1.25, 2.5, 5, and 10 μM) for 0.5 h, (**B**) 1 h, and (**C**) 2 h, respectively. (**D**) 7-dpf transgenic zebrafish larvae were treated with 12.5 μM of neomycin only for 0.5 h or pretreated with 5 μM CA for different times (0, 0.5, 1, 2, 3, 4, 5, and 24 h). (**E**) 7-dpf transgenic zebrafish larvae were pretreated with 5 μM of CA for 1, 2, and 3 h followed by CA washout, and subsequently 0.5 h of 12.5 μM neomycin treatment. Fluorescence micrographs of lateral line hair cells from neuromasts (O, OC, MI, PLL1, PLL3, and PLL4) (right panel) were analyzed and the quantitative result of viable hair cells was present as a percentage of the untreated control (left panel). All values of the experimental groups were presented as mean ± SD. ** *p* < 0.01, *** *p* < 0.001, and ns, no significant difference as compared with untreated control.

Next, we applied the pretreatment protocol to determine the protective ability of CA against the ototoxic effects of neomycin. The 7-dpf transgenic zebrafish larvae were pretreated with 1.25, 2.5, 5, and 10 μM of CA for the indicated times (0.5, 1, and 2 h) and then co-treated with 12.5 μM neomycin for 0.5 h. Compared with the control, 12.5 μM neomycin only and pretreatment with serial concentrations of CA (1.25, 2.5, 5, and 10 μM) for 0.5 or 1 h resulted in a significant decrease in the number of hair cells (*p* < 0.05) (Figure 2A,B). Notably, transgenic larvae pretreated with 5 μM of CA for 2 h exhibited significantly increased hair cell viability (*p* < 0.05) (Figure 2C). Thus, we further assessed the time-course response of 5 μM CA from 0–24 h in ameliorating neomycin-induced hair cell damage. The result indicated that the viability of hair cells increased significantly at 2 h and 3 h compared to the untreated control (*p* > 0.05). However, the protective effect of CA diminished with time. After 4–5 h in CA pretreatment, its protective efficacy was only 70% compared with the control group. Under 24 h CA pretreatment, the viability of hair cells was found to be significantly decreased to a level similar to that of neomycine alone. Therefore, the promising otoprotection of 5 μM CA was demonstrated to exist at 2 and 3 h pretreatment of CA (Figure 2D). To characterize whether protective effects still persist after washout and removal of CA prior to neomycin exposure, 7-dpf transgenic larvae were pretreated with the concentration of 5 μM of CA for 2 h, and then washed extensively before exposure to neomycin. The result showed that after washout of 5 μM CA, the hair cell viability of transgenic larvae was significantly decreased, as well as the neomycin treated group (Figure 2E).

### 2.4. The Effects of CA on Apoptosis after Neomycin Treatment

**Figure 3:**
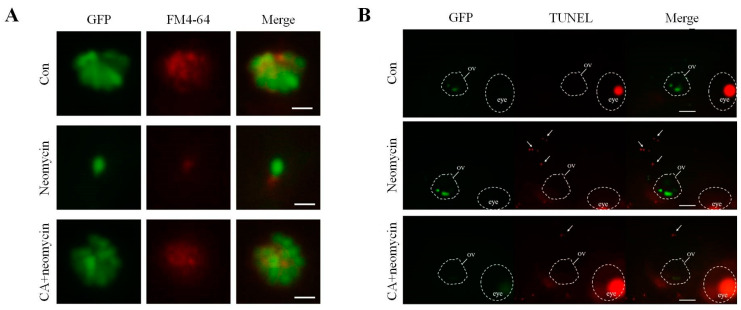
Evaluation of CA protected against neomycin-induced apoptosis and mechanotransduction channel impairment. (**A**) Fluorescence photograph of hair cells in neuromasts of 7-dpf transgenic zebrafish larvae treated with 12.5 μM of neomycin for 0.5 h only or pre-treated with 5 μM CA for 2 h. Labeling FM4-64 fluorescence dye that passes through hair’s h mechanotransduction channel was market d as a red-color signal. A comparison of the signal intensity between untreated control, neomycin, and neomycin pre-treated with CA for 2 h showed that CA prevented neomycin-induced mechanotransduction channel impairment. Scale bar: 10 μm. (**B**) Apoptotic hair cells were marked as light-respotsot (middle le) in the anterior region of the lateral line system after TUNEL staining. The location of the oval window (ov) and eye a were marked by the white circle. Comparison of TUNEL-positive signal intensity between untreated control, neomycin, and neomycin pre-treated with CA for 2 h showed that CA prevented neomycin-induced apoptosis of TUNEL-positive cells. Scale bar: 100 μm.

In situ evidence for the ability of CA to prevent hair cell loss caused by neomycin was further assessed by conducting FM4-64 staining and TUNEL assays. Microscopic evaluations with FM4-64 staining showed that after neomycin treatment, the intensity of GFP signal and FM4-64 staining of hair cells in the lateral line was significantly decreased, and pretreatment with 5 μM of CA for 2 h remarkably increased the GFP signal and FM4-64 staining (Figure 3A). A similar observation to the comparison of color intensity between the groups was that the number of TUNEL-positive cells was higher than that of the untreated control. However, 2 h with 5 μM of CA decreased the number of neomycin-induced apoptotic cell deaths of hair cells over otic vesicles (Figure 3B). These results indicate that CA effectively prevented hair cells from neomycin-induced ototoxicity.

### 2.5. Effect of CA on the Behavioral Activities of Transgenic Zebrafish Larvae

**Figure 4:**
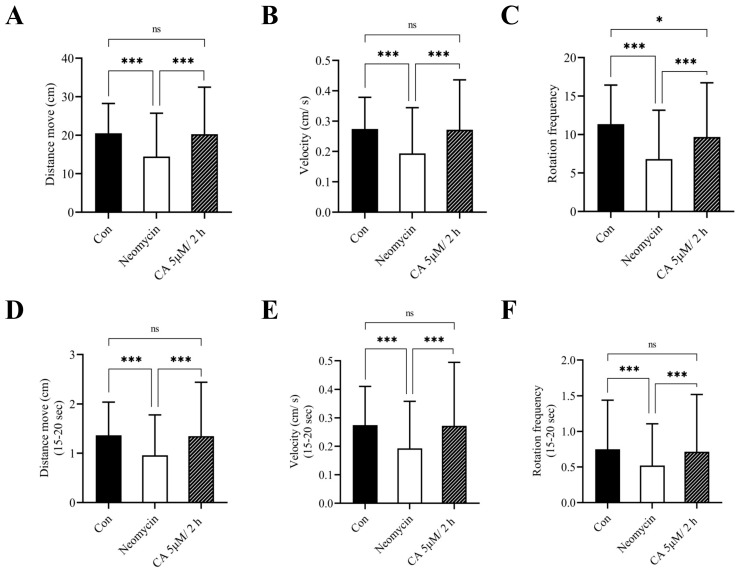
Otoprotective effects of CA on locomotor behavior of transgenic zebrafish larvae. After the exposure period, the transgenic zebrafish larvae were individually transferred into a 12-well plate and examined the behavioral parameters including (**A**) distance moved, (**B**) velocity, and (**C**) rotation frequency in response to sound and vibration stimulus caused by the tapping device. Short-time (**D**–**F**) escapes response within 15 s were confirmed and consisted of (**A**–**C**). All values of experimental groups were presented as mean ± SD. * *p* < 0.05, *** *p* < 0.001 and ns, no significant difference as the comparison between indicated group.

To corroborate the otoprotective effects of CA on hair cells, we assessed the escape response of the larvae governed by distance traveled, the number of total rotational movements, and swimming speed after tapping vibration was applied. Compared to the untreated control, the behavioral parameters, including distance moved, velocity, and rotation frequency were significantly decreased after treatment with 12.5 μM neomycin for 0.5 h, with changes that were marked reversed with 2 h of 5 μM CA pretreatment (Figure 4A–C). Moreover, the results of a 5-s (15–20 s) short-time escape response test showed that the behavioral activities of these larvae were obviously influenced by the presence of neomycin and were markedly reversed by CA pretreatment (Figure 4D–F).

## 3. Discussion

Neomycin, an aminoglycoside antibiotic, is characterized by an aminocyclitol group that exerts a broad spectrum of antibacterial properties. However, the clinical side effect of ototoxicity limits the use of aminoglycoside antibiotics, drugs considered the leading preventable cause of hearing loss. The excess production of ROS is believed to be an early event implied in the development of aminoglycoside-induced ototoxicity, which eventually leads to apoptotic cell death. Emerging efforts have been documented to prevent and treat ototoxic hearing loss. Antioxidant and anti-free radical agents that protect against the ototoxic effect of aminoglycosides have attracted increasing interest and have been studied both in vivo and in vitro [27,28,29]. CA, a hydroxycinnamic acid, can exert various potent bioactive functions through its radical scavenging actions and offers other health benefits attributed to the esterification of its hydroxyl group, which confers its free radical scavenging property [30]. In the present study, we assessed the protective efficacy of CA against neomycin-induced hair cell injury and first described its clinical applicability in suppressing the ototoxic effects caused by neomycin in an in vivo model of transgenic zebrafish.

Although naturally occurring phenolic compounds exhibit great pharmacotherapeutic efficacy, the dose-dependent toxic effect of CA must be determined with the intention of reducing undesirable properties [31,32]. Herein, CA concentrations ranging from 0–30 μM were assessed with time-dependent analysis. Aarland et al. introduced a fundamental principle of bioavailability criteria and indicated the potential toxicity of CA generated by two catechol functional groups [33,34]. CA-rich extracts from *Echinacea purpurea* have been demonstrated to exhibit cytotoxic effects via activating caspase -3/-7 mediated apoptotic pathway in a time- and dose-dependent manner on MIA PaCa-2 (IC_50_:62.92 ± 2.24 μg/mL) and COLO320 (IC_50_: 25.36 ± 1.14 μg/mL) human cancer lines [35]. In this study, zebrafish larvae were exposed to up to 20 μM (9.5 μg/mL) of CA to further explore their survival and their protective capacity. The superficial location of neuromast hair cells within the head and mechanosensory lateral line system of zebrafish allows for rapid in vivo ototoxic evaluation with short-period exposure to neomycin and potential ototoxicity protection candidates [36,37,38]. As there is no obvious difference in the susceptibility to aminoglycosides between neuromasts, the average number of hair cells counted from the anterior (O, OC, and MI) and posterior (PLL1, PLL2, and PLL3) lateral line regions was assessed for acute toxic reactions in transgenic zebrafish strain Tg (*pvalb3b*: *TagGFP*) in the present work. We found that either 10 μM or 20 μM of CA treatment rapidly induced a significant reduction in hair cell numbers within 1 h, which was consistent with the acute toxicity test of zebrafish larvae viability. Moreover, to assess whether CA could significantly prevent neomycin-induced hair cell loss, the larvae were pretreated of 0–10 μM of CA and 12.5 μM neomycin simultaneously for 0.5 h. As expected, neomycin treatment resulted in a significant loss of neuromast hair cells. Although pretreatment with 1.25, 2.5, and 10 μM CA for 2 h resulted in an increase in viable hair cell numbers in neomycin-treated larvae, no statistical difference was observed. Intriguingly, the otoprotective efficacy (~80%) was observed in 2 h of the 10 μM CA pretreatment group, which somehow differs from the previous acute toxic experiment. We speculated that it might be partly attributed to the potential toxicity of CA, and those data provide a novel founding and potentially useful information for further pharmacological research on CA.

Next, pretreatment with 5 μM of CA for 2 and 3 h effectively protected the larvae against neomycin-induced hair cell loss, with viability close to levels in the untreated control. Liu et al. reported that CA significantly ameliorated BV-2 microglial cell damage caused by lipopolysaccharide, improved mitochondria functions, and reduced MAPK/NF-κB-mediated mechanical stress. Administration of water extract from *Moringa Oleifera* protected against aminoglycoside-induced hair cells loss and reversed mitochondrial membrane potential of Corti explants through suppressing ROS production [39]. Protective actions exerted by stimulating Keap1/Nrf2 transcription activity resulted in an antioxidant defense capacity that ultimately restored the intracellular redox status in a C57BL/6J mice model [40]. Promising hepatoprotective activity of CA was also observed in methotrexate-induced hepatotoxicity in Wistar rats. Pretreatment with CA increased the liver gene expression of Nrf2, HO-1, and PPAR-γ, as well as ameliorated ROS- and lipid peroxidation-generated oxidative stress. This hepatoprotective action was also partially due to the anti-apoptotic property of CA [41]. Moreover, Liu and colleagues conducted a comparative study of the bioactivities of CA and its metabolites generated by liver microsomes. Results from liquid chromatography-mass spectrometry analysis showed that major compounds of CA hepatic phase I metabolites, including caffeic acid and caftaric acid, had great free-radical scavenging abilities against DPPH, OH, and ABTS and ferric reducing power in 3T3-L1 preadipocytes. Notably, among these three compounds, CA exhibited the most powerful scavenging activity within a certain dose range. Collectively, the aforementioned studies indicated a ROS and free-radical scavenge role for CA both in vivo and in vitro. Here, we observed that transgenic larvae pretreated with 5 μM of CA for 2 and 3 h effectively prevented neomycin-induced hair cell loss during the time course of 5 h exposure. Although the statistical difference was shown in the 4- and 5-h CA pretreated group when compared to the control, some protection (~70%) was found at those time points. In addition, based on the aforementioned results, we further assessed the sustained otoprotective efficacy of CA (5 μM) after drug washout. Intriguingly, 1–3 h pretreatment with the optimal concentration of CA (5 μM) followed by washout leads to loss promising protective effect on hair cells of neomycin-induced hair cells loss. It seems that the ideal condition of CA-mediated otoprotective action in the presence of CA is necessary. Wang et al. applied high-performance liquid chromatography coupled with a tandem mass spectrometry (HPLC-MS/MS) analytic system to explore the pharmacokinetics and tissue distribution of CA in Sprague Dawley rats by gastric feeding of 50 mg/kg body weight CA. The results from pharmacokinetics parameters revealed that the mean elimination half-life of CA was 4.5 h in rat plasma samples [42]. This might explain the otoprotective effects peaking at 2 and 3 h following sustained decreased and lost promising protective efficacy at 24 h. The excess production of ROS is considered a trigger initiating irreversible program cell death via intrinsic caspase-dependent and extrinsic receptor-mediated pathway [43,44]. Indeed, the extrinsic cell death pathway has been widely accepted to mediate the aminoglycoside-driven apoptosis in hair cells [45]. According to our previous aminoglycoside-induced hair cell damage, neomycin significantly stimulated ROS production and activated program cell death in Tg transgenic zebrafish, considered a crucial target mechanism of otoprotective strategies. The ototoxic effects of neomycin have also been reported to involve c-Jun N-terminal kinase (JUN) and caspase-9 signal transduction [46,47]. Results from in vitro model of cochlear organotypic culture revealed that gentamicin dose-dependently induced outer and inner hair cells damage mediated by increasing ROS and reduced mitochondrial bioenergetics, which was attenuated via inhibition of apoptotic pathway [13]. The apoptotic hair cells of neuromasts were assessed using TUNEL analysis to verify whether neomycin-induced hair cell injury of the posterior lateral line might be effectively prevented by CA treatment. The results revealed that neomycin induced apoptotic cell death and the hair cell loss was distributed in the lateral line. The increased light-red signals in the whole neuromasts were considered evidence of apoptotic reaction in neomycin-induced toxicity, and CA significantly decreased the TUNEL positive signal, indicating that hair cell apoptosis caused by neomycin was effectively prevented by CA treatment in transgenic zebrafish larvae. Other side effects of aminoglycosides treatment were also reported in a rodent model. Systemic studies demonstrated that gentamicin-induced nephrotoxic damage leads to apoptotic cell death in renal tissue. The main reason is related to triggering the oxidative-inflammatory cascade. Natural bioactive compounds or extracts, such as Naringin and *Descurainia sophia* extract, were also reported to have renoprotective effects on gentamicin toxicity through modulation of apoptotic renal injury in rats [48,49]. Further, aminoglycoside passes through the cytoplasm of hair cells mainly through the mechanotransducer (MET) and acts as a MET channel permeant blocker, leading to the formation of an electron acceptor that generates ROS accumulation [37,50]. Thus, we performed vital dye FM4-64 staining to visualize and verify the impact of neomycin on the MET channel. We observed that neomycin treatment reduced the loading of FM4-64 dye into the hair cells of zebrafish, a mechanotransduction impairment outcome that was effectively prevented by CA. These results emphasize the preventive properties of CA against apoptosis induced by neomycin ototoxicity in hair cells. In addition to oxidative damage, neomycin ototoxicity is related to fish mechanosensory system ablation, which contributes to significant behavioral impairment [36,51,52]. The behavior of transgenic zebrafish larvae was analyzed using end-point parameters of locomotor performance, including distance moved, velocity, and rotation frequency, to assess the protective effect of CA on fish behavior. Here, we observed a clear correlation between neomycin treatments and apoptotic hair cell injury and worsened locomotor activities in transgenic zebrafish larvae. Notably, pretreatment with otoprotective CA before neomycin significantly rescued behavioral alterations. Previously, mechanosensory-mediated behavioral alterations in zebrafish have been correlated with toxic drug exposure [53,54]. Stably established locomotion in zebrafish and high homologous genetic conservation with homo species make it a suitable tool for assessing ototoxic responses to environmental pollutants [38,55]. Although such studies have yielded insight into the chemical ablation of the mechanosensory lateral line system significantly altered locomotor activities as well as the mechanical understanding of apoptotic hair cell damage, the protective effects in fish behavior have not been fully clarified. For the first time, we established a high-throughput method for evaluating locomotor alteration in zebrafish by assessing the effects of an otoprotective medication on locomotor parameters prior to toxic agent treatment and exhibiting the relative results of hair cell damage.

## 4. Materials and Methods

### 4.1. Zebrafish Preparation and Maintenance

A transgenic strain Tg (*pvalb3b*: *TagGFP*) of zebrafish was used in this study as described elsewhere [26]. The adult zebrafish were maintained under recirculating aquaculture system of 14 h light/ 10 h dark cycle environment at 28 °C. The embryos were cultivated within water buffered with sea salt (0.06%) at 28 °C. The experimental manipulations involving animals were in accordance with Animal Care and Use Committee and review by Laboratory Animal Center of Chung Shan Medical University (IACUC No: 2040).

### 4.2. Acute Toxicity Test

To evaluate the acute toxic effect of CA, seven-day post-fertilization (dpf) transgenic zebrafish larvae (n = 30/group) were treated with CA (Sigma-Aldrich, St. Louis, MO, USA) at indicated concentrations (0, 1.25, 2.5, 5, 10, 20, and 30 μM) for 0.5 h, 1 h, and 2 h, respectively in a culture oven at 28.5 °C. After exposure to the period, the viable transgenic zebrafish larvae were determined under an inverted fluorescence microscope (Olympus CKX53, Tokyo, Japan) and results were expressed by comparing to them the untreated control.

Additionally, different protocols were carried out to evaluate the cytotoxic and protective effect of CA on hair cells. For the cytotoxic activity test, 7-dpf transgenic zebrafish larvae were treated with CA at indicated concentrations (0, 1.25, 2.5, 5, 10, and 20 μM) for 0.5 h and 1 h, respectively, in a 6-well plate. Additionally, the 7-dpf larvae were firstly treated with 0, 1.25, 2.5, 5, and 10 μM CA for indicated times (0.5, 1, and 2 h, respectively), and then simultaneously treated with neomycin (Sigma-Aldrich, St. Louis, MO, USA) for 0.5 h by adding neomycin stock solution to achieve a concentration of 12.5 μM. After the exposure period, the larvae were then washed twice with the embryo medium followed by anesthetizing the larvae with 0.4% tricaine (Merck KGaA, Darmstadt, Germany) for 5 min and mounted on a glass slide with 3% methylcellulose (Sigma-Aldrich, St. Louis, MO, USA). For quantitative analysis, numbers of hair cells within the otic (O), occipital (OC), middle (MI), and posterior lateral line (PLL1, PLL3, and PLL4) of neuromasts were determined and photographed using an inverted fluorescence microscope (Olympus CKX53, Tokyo, Japan).

### 4.3. Washout Test

A washout protocol was performed to evaluate the sustained protective efficacy of CA after drug washout. The 7-dpf transgenic zebrafish larvae were pretreated with 5 μM of chronic acid for 1, 2, and 3 h, respectively. After CA washout, the larvae were sequentially treated with 12.5 μM neomycin for 0.5 h. After the exposure period, the larvae were then washed twice with embryo medium, followed by anesthetizing the larvae with 0.4% tricaine for 5 min and mounted on a glass slide with 3% methylcellulose. For quantitative analysis, numbers of hair cells within the aforementioned anterior and posterior-lateral lines neuromasts were determined and photographed can use an inverted fluorescence microscope.

### 4.4. FM4-64 Staining

To evaluate the protective effect of CA on neuromast hair cells within the posterior lateral line, the hydrophilic dye, FM4-64 (N-(3-Triethylammoniumpropyl)-4-(6-(4-(Diethylamino) Phenyl) Hexatrienyl) Pyridinium Dibromide) (Thermo Scientific, Rockford, IL, USA), was carried out to determine the need for living cells. The 7-dpf transgenic zebrafish larvae were treated with neomycin for 0.5 h with or without CA (5 μM, 2 h) pretreatment. After the exposure period, the larvae were incubated in an embryo medium containing 50 μM FM4-64 dye for 10 min and then wash twice with a fresh embryo medium. Anesthetized with 0.4% tricaine and embedded in 0.5% low melt agarose on a glass slide, they were determined and photographed with an inverted fluorescence microscope (Olympus CKX53, Tokyo, Japan).

### 4.5. TUNEL Assay

The extent of apoptotic neuromast hair cells within the posterior lateral line was determined using the terminal deoxynucleotidyl transferase dUTP nick-end labeling (TUNEL) methods with an in situ cell detection kit, TMR, Red (Roche Diagnostics GmbH, Mannheim, Germany) according to the manufacturer’s instruction. Briefly, the 7-dpf transgenic zebrafish larvae were treated with neomycin only or CA pre-treatment and fixed in 4% paraformaldehyde overnight at 4 °C. The larvae were then incubated with 50 μL of TUNEL reaction mixture (TdT and fluorescein-dUTP) for 1 h in a 37 °C humid atmosphere. The intensity of TUNEL staining within posterior lateral line neuromasts was determined and photographed using an inverted fluorescence microscope (Olympus CKX53, Tokyo, Japan).

### 4.6. Behavioral Analysis

The larvae locomotor activity was performed by conducting a DanioVision observation chamber and video tracking software (EthoVision XT) was used for zebrafish behavioral analysis. After the exposure period, the larvae were individually transferred into a 12-well plate and we examined the behavioral parameters including distance move, velocity, and rotation in response to sound and vibration stimulus caused by the tapping device. The protocols of the trials were set to start when the center-point of the fish is in Arena and given an intensity level of 8 vibratory pulses every 15 s that lasted for a total of 75 s. The 12-well plate was placed in a dark-field observation chamber and the spontaneous locomotion was generated by a high-speed IR-sensitive digital GigE camera that recorded from each well of individual representative larvae. Fish behavior was quantified with EthoVision XT software, generating each representative trace.

### 4.7. Statistical Analysis

All experiments were performed in triplicate and the values are presented as mean ± standard deviation (SD). Statistical significance within groups was determined using one-way ANOVA followed by Tukey’s post hoc test. The criterion for the statistical difference was considered only if the *p*-value under 0.05 (* *p* < 0.05, ** *p* < 0.01, and *** *p* < 0.001).

## 5. Conclusions

To the best of our knowledge, the present work is the first study to assess the otoprotective efficacy of CA on neomycin-induced toxicity in the hair cells of transgenic zebrafish (*pvalb3b*: *TagGFP*), which appears to be a unique model for ototoxic hearing loss. Our results identified the otoprotective role of CA against neomycin-induced apoptotic hair cell death in lateral line neuromasts. In situ evidence also confirmed the otoprotective efficacy of CA in ameliorating neomycin-mediated program cell death and mechanotransduction machinery impairment. The novelty of this research study is that we developed a high-throughput analysis that can provide information about behavior alternation in response to different medications, and might potentially be used as a chemical screening platform for investigating the ototoxic hearing loss. Therefore, at appropriate concentrations, CA may have a potential otoprotective effect against neomycin-induced ototoxicity. Combining the above results will allow us to develop potential synergistic otoprotective drugs containing CA to prevent hair cell damage caused by ototoxic drugs.

## Data Availability

Not applicable.
